# Editor's Letter-Box

**Published:** 1899-09-16

**Authors:** 


### EDITOR'S LETTER-BOX.

The Dean of the Medical Faculty, University College, Dundee, writes to say that the whole course for the medical degree may be taken out at the college. Two *anni medici* are provided at St. Andrews and the whole five at Dundee. He also says that University College is "one of the constituent colleges of the University of St. Andrews," whereas we described it as "connected with the University of St. Andrews," for which we are duly sorry.

